# Nurses’ attitude and perceived barriers to pressure ulcer prevention

**DOI:** 10.1186/s12912-018-0282-2

**Published:** 2018-04-16

**Authors:** Werku Etafa, Zeleke Argaw, Endalew Gemechu, Belachew Melese

**Affiliations:** 1 Department of Nursing, College of Health Science, Wollega Unversity, Samara, Ethiopia; 2 School of Nursing, College of Health Science, Addis Ababa University, Addis Ababa, Ethiopia; 3 Department of Statistics, College of Natural and Computational Sciences, Arsi University, Asella, Ethiopia

**Keywords:** Wound, Pressure ulcer prevention, Nurses attitude, Perceived barrier

## Abstract

**Background:**

The presence or absence of pressure ulcers has been generally regarded as a performance measure of quality nursing care and overall patient health. The aim of this study- wasto explorenurses’ attitude about pressure ulcer prevention’and to identify staff nurses’ perceived barriers to pressure ulcer prevention public hospitals in Addis Ababa, Ethiopia.

**Methods:**

A self-reported multi-center institutional based cross sectional study design was employed to collect data from staff nurses (*N* = 222) working in six (6) selected public hospitals in Addis Ababa, from April 01–28/2015.

**Results:**

Majority of the nurses had (*n* = 116, 52.2%) negative attitude towards pressure ulcer prevention. The mean scores of the test for all participants was 3.09out of 11(SD =0.92, range = 1–5). Similarly, the study revealed several barriers need to be resolved to put in to practice the strategies of pressure ulcer prevention; Heavy workload and inadequate staff (lack of tie) (83.1%), shortage of resources/equipment (67.7%) and inadequate training (63.2%) were among the major barriers identified in the study.

**Conclusions:**

The study finding suggests that Addis Ababa nurses have negative attitude to pressure ulcer prevention. Also several barriers exist for implementing pressure ulcer prevention protocols in public hospitals in Addis Ababa, Ethiopia. Suggestion for improving this situation is attractive.

## Background

Pressure ulcers are defined as localized injury to the skin and/or underlying tissue usually over a bony prominence, as a result of pressure, or pressure in combination with shear [1]. PUs significantly limits many aspects of an individual’s well-being, including general health and physical, social, financial, and psychological quality of life [2]. In United States nearly 1 million people develop pressure ulcers annually, while approximately 60,000 acute care patients die from related complications [3]. The estimated cost of managing stage III/IV pressure injury per patient is $70–150 thousand, and the total cost for treatment of pressure ulcers in the United States is estimated at $9–11 billion per year [4].

Research evidences displayed that Pressure ulcer prevalence is varying from country to country For example, prevalence of pressure ulcer in Jordan (12%), Nigeria (3.22%), (Norway, 17%, Irish, 16%, Denmark, 15%, Sweden, 25%), Irish (9%), (Norwegian, 54% & Irish, 12%), Wales (8.9%) [5–10].

One study [11] identified risks for the development of pressure ulcers/injuries included advanced age, immobility, incontinence, inadequate nutrition and hydration, neurosensory deficiency, device-related skin pressure, multiple comorbidities and circulatory abnormalities.

A systematic review reported that pressure ulcer incidence rates vary considerably by clinical setting; ranging from 0.4 to 38% in acute care, from 2.2 to 23.9% in long term care, and from 0 to 17% in home care [12]. A retrospective secondary analysis of database studies have shown that an estimated 3.5–4.5% of all hospitalized patients are developing potentially preventable, hospital-acquired pressure ulcers, despite heightened awareness [3]. Hospital-acquired pressure ulcers/injuries (HAPU/I) result in significant patient harm, including pain, expensive treatments, increased length of institutional stay and, in some patients, premature mortality [13].

A single published study by Haileyesus & Mignote [14] conducted in Ethiopia in Felegehiwot referral hospital, among 422 found the overall prevalence rate of 16.8%. Of this, 62%, 26.8% and 2.8% developed stage I, II and stage IV pressure ulcer, respectively, based on European Pressure Ulcer Advisory Panel (EPUAP). This research also reported that the significant variables with the presence of PU such as stay in hospital for a long, slight limit of sensory perception, and friction and shearing forces.

Fishbein & Ajzen [15] explicated that attitude is learned and is affected by knowledge, behavioral intent and the amount of affection for or against an object. Aperson who holds a positive attitude toward an issue will have a greater possibility of performing a supportive behavior related to that issue [15]. For example, the more positive attitude of nurses to PU prevention, the better practice of PU prevention care demonstrated [16].

Evidence-based clinical guideline has a significant correlation with positive feeling to pressure ulcer prevention [17]. Grimshaw et al. [18] stated that lack of knowledge, negative attitudes, or underdeveloped skills are the principal barriers to evidence-based practice at the level of the individual health care professional. Ayello & Meaney [19] also explicated negative attitude of nurses to PU prevention increase the prevalence rate of pressure ulcers. Similarly, Hill [20] expressed that nurses’ negative attitude could be affected by shortage of staff, lack of time, lack of knowledge and insufficient equipment.

Among the researched and published documents on the same topic, six studies concluded that most nurses hold a positive attitude to PU prevention (Moore & Price 2004, Kallman & Suserud 2009, Islam 2010, Demarré et al. 2011, Tubaishat et al. 2013, and Uba et al. 2014) [21–26]. In addition to attitude of nurses explored, three papers identified the major barriers for nurses’ to demonstrate PU prevention practice such as lack of time, staff and uncooperative patient [21, 22, 25].

However, a study conducted among 145 Belgian nursing homes by Beeckman et al. [17] using convenience sampling found that poor attitude to PU prevention. Similarly, another data collected from 105 health care professionals (nurses, physical therapist, occupational therapist and physician medicine) in the rehabilitation at Fahad Medical College city, Riyadh found unsatisfactory attitude of health care professionals to PU prevention [27]. A cross sectional study among Jordanian nurses also found a positive relationship between positive attitude of nurses and longer year of experience [25].

Pressure ulcer prevention is a priority for nurses, healthcare professionals and healthcare organizations throughout the world, and a key factor in pressure ulcer prevention and management is individual nurse decision making [28]. Nurses hold the most responsibility for prevention and management of pressure ulcers though it is a multidisciplinary team approach [29].

Padula et al. [3] described that hospitals adhering to PU updates had significant pressure injury reductions by average hospital 7.5 pressure injury case reductions and $500,000 + savings per year. Moore & Price [21] suggested that pressure ulcer prevention and management involves both emphasizing on educational strategies and promoting a positive attitude of nurses towards PU care.

To date, no similar studies have been conducted in Ethiopia to examine nurses’ attitude and perceived barriers to PU prevention. Therefore, this study was undertaken to assess attitude of nurses in Public Hospitals in Addis Ababa to PU prevention.

### Objectives

The objective of this study was to explore nurses’ attitudes toward the prevention of pressure ulcers, and to identify staff nurses’ perceived barriers to pressure ulcers prevention in Public hospitals in Addis Ababa, Ethiopia.

## Methods

### Study design

Institutional based cross sectional multi-center study using quantitative method was employed from April 01–28, 2015.

### Study setting and sample

The study was in Addis Ababa, the capital city of Ethiopia which contains 13 public referral hospitals (each contains from 120 to 400 beds for admission). There are 34 private hospitals, 86 health centers and various NGOs and health institutions. The data in this study included nurses working from patient admission units in six randomly selected public referral hospitals (46%). The units included were medical, surgical, orthopedics, intensive care unit, gynecology, pediatrics, dermatology, burn and oncology.

### Sample size and sampling procedure

The sample size was determined using a formula of estimating a single population proportion for cross sectional study. Since the population size is less than 10, 000 (*N* = 534), the final sample size was estimated using correction formula. The final sample size obtained including 10% non-response rate was 252. Then, the number of participants in each selected hospitals to obtain similar proportion of participants were determined using the population proportionate sampling (PPS). It is estimated using the formula: n = (nf * N in a health facilities)/N _total,_ where, n = Proportion of nurses participate in the study in a given public hospital, nf = Final sample size obtained using correction formula (252)_,_ N = is the total number of nurses in the selected public hospitals (534).

### Study instrument

A questionnaire used for gathering data contained three parts. For the purpose of the current study, demographic information which may or may not have an impact on the nurses’ attitude towards pressure ulcer prevention (age, sex, clinical working experience, educational level, and the nurses received training on PU prevention and read research articles about it) were added.

Part two of the data collection tool was Pressure Ulcer Attitude Test tool contained 11 statements developed and validated by Moore & Price [21]. In this section, the response option utilized a 5 point Likert scale from strongly disagree to strongly agree. It was chosen since it allows scaling of an individual’s attitude and is more sensitive to the full range of attitude than a simple dichotomous agree/disagree option. The validity of instrument were assessed by nursing instructors holding MSc (Assistant professors) and had research experience (*n* = 3) before and after pilot study.

Piloted test was conducted at St. Peters hospital Research after Review Ethical Committee granted us a letter of permission. After pilot test, marginal corrections such as order and wording of questions were assessed. Similarly, the questionnaire was pilot tested (*n* = 25). The internal consistency reliability (Cronbach’s α) was 0.76.

Part three of the data collection tool in the questionnaire was comprised a closed-ended questions (‘Yes’ or ‘No’ response) to identify nurses’ barriers to implement pressure ulcer prevention protocol adapted by reviewing different literatures [21, 22, 25].

**Fig. 1:**
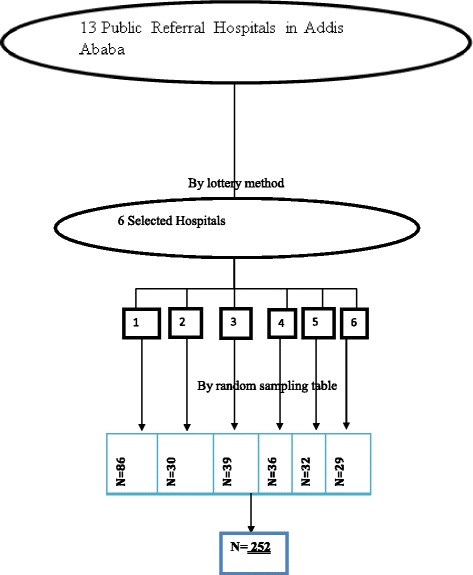
Schematic Presentation of Sampling Procedure

The hospital which agreed for participation was asked to give the list of their participants through matron. The head nurses at study site were asked for their assistance to distribute questionnaires and were cooperative. The participants were selected using random sampling table (Fig. 1).

### Data analysis

The data cleaning was done, entered in to computer using EPI data version 3.1 statistical packages, and 10% of the response was randomly selected and checked for the consistency of data entry. SPSS version 20 was used for data analysis. Frequencies and percentages were calculated to all variables which were related to the objectives of the study. The mean score attained from the scale was used to measure nurses’ attitude. A numeric value was assigned for each attitude test items: 5 = strongly agree, 4 = Agree, 3 = neither agree nor disagree, 2 = disagree, and 1 = strongly disagree. The questions include both positive and negative statements. But for negatively stated questions the score is reversed. The attitude mean was obtained by collapsing the Likert scales strongly disagree, disagree and neither agree nor disagree to the negative attitude, and strongly agree and agree to the positive attitude. Appropriate inferential test like ANOVA (analyses of variance) were used to test the effect of demographics on attitude. Results for *p*- value < 0.05 were considered significant.

## Results

### Demographic characteristics of the nurses

A total of 252 professional nurses were invited to participate in the study, 222 fully participated in the study, for a response rate of 78.7%. Among 369 nurses 128 (36%) were males. The mean ages of participants were 29 with minimum 20 and 61 years maximum. Most participants had a bachelor’s degree in (*n* = 140, 63%), while 11% (*n* = 24) were enrolled in masters of Science degree in nursing. Nurses who are counted for their experiences in more than 10 years were 20.2% while majority of them 54% have 1–4 years of experience in nursing profession. Sixteen nurses (*n* = 16, 7.2%) reported that they had received and the largest proportion of them (*n* = 148, 66.7%) never received any training in PU prevention, while majority of them (*n* = 191, 86%) had not previously read research articles about PU compared to 31 (14%) who had read it. A limited number of nurses had attended PU training on conference. The majority of the participants were from medical ward (30.0%) as shown in Table 1.

**Table 1:** Frequency distribution of nurses’ socio-demographic variables (*N* = 222)

Variables	*N* (%)
Sex	
• Male	77 (34.7)
• Female	145 (65.3)
Age (M = 29, SD = 6.65,max = 61,min = 20)	
• 20–29 years	148 (66.7)
• 30–39 years	49 (22)
• > = 40 years	25 (11.3)
Level of education	
• Diploma in nursing	58 (26)
• Degree in nursing	140 (63)
• Masters in nursing	24 (11)
Working experience (max = 41, min = 1)	
• 1–4 years	115 (51.8)
• 5–10 years	55 (24.8)
• Above 10 years	52 (23.4)
Where you received training on PU prevention?	
• In-service	16 (7.2)
• Course	37 (16.7)
• Conference	2 (0.9)
• Workshop	19 (8.5)
• Never	148 (66.7)
Have you read researchs about pressure ulcers?	
• Yes	31 (14)
• No	191 (86)

### Nurses’ attitude towards pressure ulcer prevention

The study result indicated that more than half (*n* = 116, 52.2%) of nurses’ attitude towards pressure ulcer prevention were negative (mean = 3.09, SD = 0.92, range = 1–5). The lowest possible score (negative attitude) was 11 whilst the highest possible score (positive attitude) was 55.

Data analysis of the nurses’ attitudes showed some interesting points in relation to certain statements (Table 2). More than half of staff nurses (*n* = 126, 56.6%) felt that all patients are at risk of developing PUs, and around three quarter of the participants (*n* = 162, 72.9%) thought PU treatment was seen as lesser priority than its prevention. Nurses also believed that PU could be voided (*n* = 153, 68.8%), PU prevention care was not time consuming (*n* = 129, 58%), and 69% was considered continuous assessment of patient would give an accurate process of identifying patient at risk for PU.

**Table 2:** Nurses’ attitude towards pressure ulcer prevention, 2015 (*N* = 222)

Variables	Nurses’ attitude rate				
	Strongly agree *N* (%)	Agree *N* (%)	Neither agree nor disagree *N* (%)	Disagree *N* (%)	Strongly disagree *N* (%)
All patients are at risk of developing PUs	64 (28.8)	62 (28)	46 (20.7)	28 (12.6)	22 (9.9)
PU prevention is time consuming for me	34 (15.3)	59 (26.6)	39 (17.6)	34 (15.3)	56 (25.2)
In my opinion, patients tend not to get as many PUs now days.	24 (10.8)	56 (25.2)	56 (25.2)	49 (22.1)	37 (16.7)
I do not need to concern myself with PU prevention in my job.	25 (11.3)	32 (14.4)	36 (16.2)	47 (21.2)	82 (36.9)
PU treatment is greater priority than its prevention.	37 (16.7)	23 (10.4)	17 (7.7)	27 (12.1)	118 (53.1)
Most pressure ulcers can be avoided	107 (48.1)	46 (20.7)	36 (16.2)	14 (6.3)	19 (8.7)
Continuous assessment of patient will give an accurate account of their PU risk	90 (40.6)	63 (28.4)	27 (12.1)	23 (10.3)	19 (8.6)
I am less interested in PU prevention than other aspects of care	22 (9.9)	34 (15.3)	2 6 (11.8)	46 (20.7)	94 (42.3)
My clinical judgment is better than any PU risk assessment tool available to me	34 (15.3)	31 (14)	32 (14.5)	36 (16.2)	89 (40)
In comparison with other areas of care, PU prevention is a low priority for me.	48 (21.5)	51 (22.9)	70 (31.4)	33 (14.8)	21 (9.4)
PU risk assessment should be regularly carried out on all patients during their stay in hospital	94 (42.3)	46 (20.7)	34 (15.3)	26 (11.7)	22 (10)

The only statistically significant association in this study was gender of staff nurses (*P* = 0.032). It found that male staff nurses showed that more positive attitude to PU prevention than female staff nurses. Other variables like age group, educational level, whether PU training received and reading research articles about PU had no effect on the nurses’ attitude to pressure ulcer prevention.

### Nurses’ perceived barriers for practicing PU prevention care

Among thestaff nurses participated in the study (*n* = 222), only 2% of them had not reported any challenge for preventing pressure ulcer while majority (98%) of them had reported different challenges. The most frequently cited barriers were heavy work load and inadequate staff (*n* = 185, 83.1%), shortage of pressure relieving devices (inadequate equipment and devices), (*n* = 150, 67.7%), inadequate training about PUprevention (*n* = 140, 63.22%), lack of job satisfaction (*n* = 125, 56.2%), presence of other priorities than PU (*n* = 130, 58.7%) and lack of universal guide lines (*n* = 133, 59.3%) as illustrated in (Table 3).

**Table 3:** Nurses’ perceived barriers practice to prevent pressure ulcer prevention (*N* = 222)

Nurses’ perceived barriers for preventing PU	Frequency (%)
Poor access to literature and reading facilities	110 (49.7)
Heavy workload and inadequate staff	185 (83.1)
Lack of universal guide line on prevention of pressure ulcer	133 (59.8)
Inadequate training coverage of pressure ulcer prevention	140 (63.2)
Uncooperative patients	87 (39.3)
Lack of job satisfaction in nursing profession	125 (56.2)
Presence of other priorities than pressure ulcer	130 (58.7)
Shortage of resources (equipment/resource)	150 (67.7)
Inadequate knowledge about pressure ulcer among nurses	60 (27)
Lack of multidisciplinary among staff nurses	64 (28.9)
I don’t have any challenge	4 (2)

## Discussion

The results of this cross-sectional study explored that Addis Ababa nurses’ hold a negative attitude to PU prevention. Similarly, major staff nurses’ barriers to practice PU prevention such as heavy workload/inadequate staff, shortage of resources and inadequate training about PU prevention were identified. The present research result contradicted with several other previous study results [21–26]. This may be due to this study participants’ included were from inpatient units. However, the present study result is in agreement with study conducted by Beeckman et al. [17] and Kaddourah et al. [27].

According to Moore and Price [21], the presence of barriers and obstacles (lack of time and staff, training, resources, and guideline) could prevent positive attitudes of nurses’ from being reflected in practice. So, for the current study, it can be interpreted that the major barriers identified by staff nurses to practice PU prevention such as heavy workload and inadequate staff, and shortage of resources and inadequate training about PU prevention could be the possible reasons for most nurses’ negative attitude.

The Knowledge, Attitude and Practice (KAP) model [29] explained that individual’s ability to perform actions can be influenced by certain knowledge, and attitude affects individual towards practice. Beeckman, et al. [17] suggested the more positive attitude towards prevention of PU, the more adequate preventive care patients will receive. This is supported by two other studies [18, 20]. In addition to identified barriers, for this study poor knowledge of nurses could be another possible reason for staff nurses’ negative attitude towards PU prevention.

This paper showed that male nurses hold more positive attitude than female nurses (*p* = 0.032) to PU prevention though no similar researched topic agree with this point. The current study is in line with Moore and Price (2004) [22], who found that nurses’ level of education and year of clinical working experience had no significant effect on nurses’ attitude. Although Tubaishat et al. [25] found that nurses who had more year of experience, showed more positive attitude, our study did not support it. In addition, the respondents who had received PU care training and read research articles about PU did not scored higher attitudes than their counter parts. This supported by other research results [21, 22].

However, Kallman and Suserud [22] identified perceived barriers such as lack of time and un-cooperative patients, and lack of pressure relieving devices as the possible barriers, whereas as, Tubaishat et al. [25] identified as lack of policies and guidelines about PU prevention (50%), lack of cooperation with other health professionals (51%) and lack of job satisfaction (57%) as the major barriers to prevent PU cited by most of the nurses. Similarly, this study displayed heavy workload and inadequate staff (lack of time) as the major barrier for being practicing PUP care, whilst, uncooperative patients as not cited as a major barrier to PU prevention. But, majority (58%) of them believed that PU is not consuming. This could be they had not sufficient time and adequate man powerto provide PU prevention.

This study described that 70.7% of nurses believed that their clinical judgment is better than risk assessment tool. This indicated they can assess PU clinically better than using risk assessment tool. Bergstrom et al. [30] found that risk assessment tool is more accurate and reliable than clinical judgment to who are at risk for PU development. However, Samuriwo, & Dowding [28] indicated that assessment tools were not routinely used to identify pressure ulcer risk, and that nurses rely on their own knowledge and experience rather than research evidence to decide what skin care to deliver.

Almost three quarter (74.8%) of the respondents also more interested in PU prevention than other aspects of nursing care. This is in line with Moore and Price study result [21] and Kaddourah et al. [27]. This suggested nurses had high interest in PU care; but, priority was given to other illnesses. This is why most of the staff nurses (*n* = 130, 58.7%) complained priority for other illnesses rather than PU as a barrier.

A significant number of the staff nurses (66.7%) surveyed had received no training to PU prevention, 191 (86%) have not ever read research about PU while 133 (59.8%) identified lack of universal guide line among the major barriers to practice prevent PU care. This idea is strengthened by the participants’ response for which majority of them had disagreed that patients are tends not to get as many PUs now days. Further, poor access to literatures and journals due to lack of electronic libraries near the nurse’ working units/wardswas another cited barrier to practice PU prevention. Hunt [31] stated that if nurses did not read scientific journals, they will not be able to integrate research into their practice.

From researchers’ experience in developing countries it is obvious that nursing care provided for patients are not adequate. This is highly due to shortage of resources. According to this study, one of the most commonly cited barriers was shortage of equipment/resource or facilities (67.7%) which is in agreement with the study finding among (Irish, Belgian and Jordanian nurses [21, 24, 25]. The shortage of resources in developed countries (among Belgian [24] and Irish [21] nurses) may be due to the participants were nurses who give caring at home. Lack of job satisfaction (56.2%) may be another reason behind for not practicing PU prevention care. According to Tubaishat et al. [25] lack of job dissatisfaction (25%) was also among the most commonly cited barrier. In Ethiopia,there is scarcity of pressure ulcer relieving devices which help nurses lifting patient or changing the patient position paying off the minimum energy particularly for severely ill patients in addition to time it saves.

Majority (66.7%) of the nurses that participated in this study reported that they never attended any training concerning pressure ulcersand about 133 (59.8) of participants reported lack of universal guideline for PUP. This indicates how much attention is paid to prevent PU in Addis Ababa. Padula et al. [4] stated hospitals adhering to PU updates had significant pressure injury reductions and $500,000+ savings per year. Currently evidences exhibited that prevalence of pressure ulcer is vary from country to country. This is supported by study results [5–10].

As observed from the participants’ characters only 26 (11%) were second degree holders and 58 58 (26%) were diploma holders in nursing. It is reported that educational program will improve the knowledge of PU prevention. Similarly, updating nurses’ education is the cardinal to increase nurses’ competency to help them better clinical decision maker [32]. Generally, authors noted that lack of knowledge, negative attitudes, or underdeveloped skills are the principal barriers to PU prevention [18, 19].

### Limitations

The data are from self-report questionnaires and qualitative method was not employed. But, since there is similar educational setting and resources fairly distributed to all hospitals, the result of the study can be generalizableto all nurses working from Addis Ababa region.

## Conclusions

In the current study, the attitude of most nurses towards PUP was negative. The study also identified the major barriers to carry out PUP practice: Heavy work load/inadequate staff or lack of time 185 (83.3%), Shortage of resources (equipment/resources) 150 (67.6%), Inadequate training coverage of pressure ulcer prevention140 (63%) and lack of universal guide line on prevention of pressure ulcer 133 (59.9%) are the most commonly cited barriers. Further research into nurses’ attitude to pressure ulcer is needed using structured interview questionnaire.

## Abbreviation

NGO: Non-Governmental organization
SD: Standard Deviation
SPSS: Statistical Package for Social Sciences
